# From a Co-Laborer

**Published:** 1876

**Authors:** 


*From a Co-Laborer.*—"We have but one objection to THE RECORD. It is all that we can reasonably ask or expect in a Medical journal. It is brief, pointed and exceedingly practical—the very desideratum so long felt by the busy practitioner in the South. The objection we want to name is its want of punctuality. Its great value and practical interest makes this objection ten-fold more annoying than it would otherwise be. It is with great pleasure, then, that we see in your December number the statement that the large and reliable establishment, the FRANKLIN PRINTING HOUSE, will hereafter take charge of its publication. We have the pleasure of knowing well the character and ability of this establishment, and as we have every reason to believe that the editors have done their duty in the past, and will continue to do it in the future, we shall confidently expect its punctual arrival hereafter. We hope this fact will stimulate each subscriber to do as you have requested, consider himself as a member of the finance committee, to collect and forward his own subscription, and encourage others to do likewise.

Another thought in this connection: Give more attention and space to your advertising department. It is becoming more and more important that practitioners of medicine should be posted as to first-class houses, that they may be able to advise druggists and others from whom to make their purchases, not only in the line of drugs, instruments, etc., but the names, prices, and character of all new books and publications."

